# Intraspecific trait variation alters the outcome of competition in freshwater ciliates

**DOI:** 10.1002/ece3.7828

**Published:** 2021-06-29

**Authors:** Sabine Flöder, Joanne Yong, Toni Klauschies, Ursula Gaedke, Tobias Poprick, Thorsten Brinkhoff, Stefanie Moorthi

**Affiliations:** ^1^ Institute for Chemistry and Biology of the Marine Environment (ICBM) University of Oldenburg Wilhelmshaven Germany; ^2^ Ecology and Ecosystem Modelling University of Potsdam Potsdam Germany

**Keywords:** ciliate predators, intraspecific trait variation, microalgal resource, predator trait variation, predator–prey systems, resource competition

## Abstract

Trait variation among heterospecific and conspecific organisms may substantially affect community and food web dynamics. While the relevance of competition and feeding traits have been widely studied for different consumer species, studies on intraspecific differences are more scarce, partly owing to difficulties in distinguishing different clones of the same species. Here, we investigate how intraspecific trait variation affects the competition between the freshwater ciliates *Euplotes octocarinatus* and *Coleps hirtus* in a nitrogen‐limited chemostat system. The ciliates competed for the microalgae *Cryptomonas* sp. (*Cry*) and *Navicula pelliculosa* (*Nav*), and the bacteria present in the cultures over a period of 33 days. We used monoclonal *Euplotes* and three different *Coleps* clones (*Col* 1, *Col* 2, and *Col* 3) in the experiment that could be distinguished by a newly developed rDNA‐based molecular assay based on the internal transcribed spacer (ITS) regions. While *Euplotes* feeds on *Cry* and on bacteria, the *Coleps* clones cannot survive on bacteria alone but feed on both *Cry* and *Nav* with clone‐specific rates. Experimental treatments comprised two‐species mixtures of *Euplotes* and one or all of the three different *Coleps* clones, respectively. We found intraspecific variation in the traits “selectivity” and “maximum ingestion rate” for the different algae to significantly affect the competitive outcome between the two ciliate species. As *Nav* quickly escaped top‐down control and likely reached a state of low food quality, ciliate competition was strongly determined by the preference of different *Coleps* clones for *Cry* as opposed to feeding on *Nav*. In addition, the ability of *Euplotes* to use bacteria as an alternative food source strengthened its persistence once *Cry* was depleted. Hence, trait variation at both trophic levels codetermined the population dynamics and the outcome of species competition.

## Graphical abstract

Intraspecific variation in the traits “selectivity” and “maximum ingestion rate” for their prey significantly affected the competition between two freshwater ciliate species. Exclusion of either one of the ciliates or long‐term persistence depended on which of the competitors benefited more from feeding on a less abundant algal resource of high food quality, on severe nutrient limitation that affected the food quality of the dominant algal resource, and on the ability of one competitor to use bacteria as an alternative food source. Hence, trait variation at both trophic levels codetermined the population dynamics and the outcome of species competition.

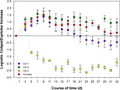

## 1 INTRODUCTION

The last two decades have seen trait‐based approaches in empirical and theoretical research complementing and in some cases even replacing traditional ones based on species identity (see, e.g., Zakharova et al., 2019). Trait‐based approaches are developed from the concept of functional groups (Cummins, 1974; Grime, 1974; Raunkiaer, 1934) that proposed to classify organisms according to their function rather than their taxonomy. A trait is defined as a distinct attribute of an organism and can be used for intra‐ and interspecific comparisons when measured at the individual or population level, respectively. Functional traits are those attributes that strongly affect the fitness of organisms (McGill et al., 2006; Violle et al., 2007) and are often clearly connected to functions at higher levels of organization (Zakharova et al., 2019). They can, therefore, be used to analyze complex dynamics of populations, communities, and food webs.

Recent trait‐based approaches have extended early concepts on competition and coexistence based on resource use traits (e.g., Grime, 1977; Tilman, 1977, 1982) by considering the many different traits that codetermine the impact of a species on the availability of limiting resources and its response to altered resource conditions (Hillebrand & Matthiessen, 2009; Suding & Goldstein, 2008). Those traits include predator specialization and maximum ingestion rate or prey edibility, and maximum growth rate (e.g., Filip et al., 2014; Tirok & Gaedke, 2010). Being more effective in either locating, capturing, or consuming resources (e.g., Egan & Funk, 2006; Norberg, 2004; Wang & Keller, 2002), specialist predators are expected to have a larger grazing impact on their prey than generalists, which are less efficient and have a wider resource spectrum. A prey species, on the other hand, may invest its energy either into defense structures or into higher maximum growth rates (Fine et al., 2006; Merico et al., 2009). Incorporating such trade‐offs into food web models has been shown to substantially influence predator–prey dynamics and biodiversity–ecosystem function relationships (Bauer et al., 2014; Tirok et al., 2011; Tirok & Gaedke, 2010). While the importance of trait variation on different trophic levels is now well recognized for ecosystem structure, functioning, and trophic dynamics (e.g., Ceulemans et al., 2020; Díaz et al., 2016; Gunderson, 2000; Hooper et al., 2005), much less is known about the potential impact of intraspecific trait variation on the structure and dynamics of food webs.

Trait variation among conspecific organisms has long been recognized (Ford, 1964; Roughgarden, 1972). There is also accumulating evidence that intraspecific trait variation may increase productivity, stability, and the likelihood for species coexistence, suggesting that intraspecific trait variation can have large ecological effects (Becks et al., 2010; Hughes et al., 2008; Klauschies et al., 2016; Raffard et al., 2019). For instance, intraspecific trait variation has been shown to markedly affect predator–prey dynamics in an experimental rotifer–microalgal system (Yoshida et al., 2003). Flöder et al. (2018) demonstrated in a microcosm experiment using ciliate predators and microalgal prey that the effect of intraspecific trait variation concerning specialization, selectivity, and grazing rate was comparable to the effect of interspecific trait variation. Differences in the feeding niches of three different clones of the ciliate *Coleps hirtus* resulted in a transgressive overyielding, that is, in a higher biomass production of the polyclonal culture compared with each of the three monoclonal cultures, including the most productive one. However, based on the lack of morphological differences of the three *C*. *hirtus* clones, it was not possible to determine the individual contributions of different clones in polyculture. Operational difficulties in distinguishing different clones of a species may explain why studies on intraspecific trait variation are rare.

In the present study, we specifically incorporated intraspecific predator trait variation to investigate how this trait variation affects the outcome of competition between two different herbivorous freshwater ciliate species, using three different clones of *Coleps hirtus* and a monoclonal culture of *Euplotes octocarinatus* (the same as used in Flöder et al., 2018).

A 33‐day chemostat experiment was conducted incubating three monoclonal and one polyclonal population of *Coleps* together with *Euplotes*, which relied on two different microalgae, *Cryptomonas* sp. and *Navicula pelliculosa* and, since none of our cultures were axenic, the accompanying bacterial community. As the clones are indistinguishable by microscopy, we developed an rDNA‐based molecular assay (based on the internal transcribed spacer (ITS) regions) to differentiate between the clones in polyculture.

Highly selective regarding microalgal prey (Flöder et al., 2018; Wilks & Sleigh, 2004, 2008), *Euplotes* was able to feed and grow only on *Cryptomonas*. However, *Euplotes* could use the accompanying bacteria as an additional resource. *Coleps* fed and grew on both microalgae, but did not survive on bacteria only. The different *Coleps* clones differed in ingestion rates on the two algae, among each other and compared with *Euplotes* (Flöder et al., 2018). While the ability to feed on both of the microalgal resources can be expected to be an advantage for the *Coleps* clones, *Euplotes* may benefit from a higher *Cryptomonas* ingestion rate compared to the *Coleps* clones and from its ability to complement its microalgal prey by bacteria.

Based on these species‐ and clone‐specific feeding traits, we expected the competitive outcome between *Coleps* and *Euplotes* to depend on the clonal composition of *Coleps hirtus* and tested the following hypotheses.

H1: *Coleps* clones mainly feeding on the preferred algal prey of *Euplotes* will be inferior to *Euplotes* as the latter can graze *Cryptomonas* more efficiently and use bacteria as an additional food source.
H2: In contrast, *Coleps* clones also feeding substantially on the alternative prey *Navicula* will coexist with *Euplotes* as none of the species has to rely on *Cryptomonas* as a sole food source.
H3: Feeding substantially on both microalgal prey species, also polyclonal *Coleps* populations, will coexist with *Euplotes*. Due to their higher trait variation, polyclonal *Coleps* populations are superior to monoclonal ones. Being able to exploit available microalgal prey more effectively, they will produce high biomass levels presumably exceeding the ones of *Euplotes*.

The experiment was complemented by a carbon budget model estimating energetic and biochemical constraints for the growth of the different ciliates.

## 2 MATERIALS AND METHODS

### 2.1 Organisms used and culture conditions

We used the freshwater ciliate predator species *Euplotes octocarinatus* (monoclonal) and three different clones of *Coleps hirtus* (*Col* 1, *Col* 2, and *Col* 3). The cryptophyte *Cryptomonas* sp. and the diatom *Navicula pelliculosa* served as prey (see Table 1 for characteristics and origin of the organisms used). Prior to the experiment, all species and clonal ciliate cultures were fed *Cry*.

**TABLE 1:** Abbreviations, origin, food preference, maximum ingestion rate (*I*_max_), average cell size, volume‐to‐carbon relationship, and carbon‐to‐volume conversion factor of algal and ciliate cultures used in the experiment. SAG: Culture Collection of Algae at Göttingen University, CCAP: Culture Collection of Algae and Protozoa; Salzburg: Dr UG Berninger, University of Salzburg, Austria. Stuttgart: Dr M Schweikert, University of Stuttgart, Germany; Pisa: Dr G. Di Giuseppe, University of Pisa, Italy; M‐D&L: Menden‐Deuer and Lessard (2000); P&S: Putt and Stoecker (1989); L‐K: Loferer‐Krößbacher et al. (1998) assuming C = 0.5·DW. Note that when adjusted for the difference in ciliate biovolume, *I*_max_ of *E*. *octocarinatus* for *Cry* is 1.72‐fold the average *I*_max_ of the *Coleps* clones

Short form	Algal and ciliate species	Origin	Food preference	Max. Ingestion rate (*I*_max_)				Average cell size (µm³)	Volume‐to‐carbon relationship	Reference	Conversion factor pg C/µm^3^
				Cells ciliate^−1^ d^−1^		pg C ciliate^−1^ d^−1^					
				Cry	Nav	Cry	Nav				
*Cry*	*Cryptomonas* sp.	SAG	/					664	$C\left[\text{pg}\right]≅0.216\cdot V{\left[\mu m^{3}\right]}^{0.939}$	M‐D&L	0.145
*Nav*	*Navicula pelliculosa*	SAG	/					100	$C\;\left[\text{pg}\right]≅0.288\cdot V{\left[\mu m^{3}\right]}^{0.811}$	M‐D&L	0.121
	*Coleps hirtus*		Cry, Nav					9,850	$C\left[\text{pg}\right]≅0.14\cdot V\left[\mu m^{3}\right]$	P&S	0.14
*Col* 1 *Col* 2 *Col* 3	*C. hirtus* clone 1 *C. hirtus* clone 2 *C. hirtus* clone 3	Salzburg Stuttgart CCAP		17.6 ± 0.44	38.8 ± 0.51	1.7 10^3^	0.47 10^3^				
				18.6 ± 0.31	12.1 ± 1.89	1.79 10^3^	0.15 10^3^				
				17.0 ± 0.64	35.1 ± 4.98	1.64 10^3^	0.43 10^3^				
*Eup*	*Euplotes octocarinatus*	Pisa	Cry	116 ± 5.4	‐‐	11.2 10^3^	‐‐	26,890	$C\;\left[\text{pg}\right]≅0.14\cdot V\left[\mu m^{3}\right]$	P&S	0.14
	Bacteria							0.05	$C\;\left[\text{pg}\right]≅0.5\cdot 0.435\cdot V{\left[\mu m^{3}\right]}^{0.86}$	L‐K	0.33

While *Coleps hirtus* is a planktonic raptorial feeder consuming bacteria, algae, flagellates, and ciliates (Buonanno et al., 2014; Madoni et al., 1990), *Euplotes octocarinatus* inhabits the benthic–pelagic interface. It is a filter feeder that is able to use suspended prey as well as mechanically detach surface‐associated bacteria and algae (Fenchel, 1986; Früh et al., 2011; Lawrence & Snyder, 1998). Although morphologically and behaviorally adapted to surfaces, *Euplotes* species will occur in the water column and feed planktonically when food supply is high (Dolan, 1991; Lawrence & Snyder, 1998). Consuming plankton is an important energy source for biofilm dwelling micropredators and can play a significant role in the trophic coupling between plankton and benthos (Früh et al., 2011; Weitere et al., 2018). Based on these findings, we expected resource competition between *Euplotes* and *Coleps* to occur in nature, making them suitable candidates for our competition experiments.

Mineral water (Volvic) was used as culture medium for our ciliate clones, while microalgae were grown in WEES culture medium (Kies, 1967). None of the cultures were axenic and free of heterotrophic flagellates. Bacterial biomass was well below 5% of the total biomass of the stock cultures. Biomass of heterotrophic flagellates was comparably low. Their abundance was deemed negligible, since it was close to or below the detection limit of our microscopical analysis. The ciliates differed in average cell size and in their feeding preferences, while microalgae differed in average cell size and edibility (Table 1). The feeding preferences of the ciliates were characterized by the trait value maximum ingestion rate (*I*_max_) (Table 1). *I*_max_ was calculated based on the data published in Flöder et al. (2018) following Frost (1972), Heinbokel (1978), and Michaelis‐Menten (see Appendix A for details). *Euplotes* feeds and grows only on *Cry*, whereas our *Coleps* clones feed and grow on *Cryptomonas* (*Cry*) and on *Navicula* (*Nav*). *I*_max_ for *Cry* and *Nav*, however, differs among the *Coleps* clones. *Col* 2 has a higher *I*_max_ for *Cry* and a lower *I*_max_ for *Nav* than the other clones. *Col* 1 and *Col* 3 show no difference in *I*_max_, neither for *Nav* nor for *Cry*.

### 2.2 Experimental setup and design

The chemostat system used consisted of 16 culture vessels (culture volume 900 ml) and corresponding medium and waste containers, tubing, and peristaltic pumps (Ismatec, Wertheim, Germany). The medium inflow and the culture suspension outflow were established via a port in the cap of the culture vessel. A compressor provided the air pressure necessary to push the culture suspension through the outflow (Del Arco et al., 2020). Magnetic stirrers were used to keep the organisms in suspension. The dilution (flow‐through) rate was 0.1 d^−1^. Experimental communities grew in a modified WC medium (Guillard & Lorenzen, 1972), which was nitrogen limited (120 µmol N/L). According to previous experiments, both microalgae grow better if organic compounds are available, which can be supplied by adding soil extract. Half (60 µmol N/L) of the N concentration in the modified WC medium, therefore, originated from a soil extract prepared following the instructions of Kies (1967). An additional 60 µmol N/L was added using the WC nitrogen stock solution (NaNO_3_). Temperature was kept constant (18°C), and illumination of the cultures vessels was from the side (100 µmol/m^2^ s^−1^ photosynthetic photon flux density), with a light‐to‐dark cycle of 12:12 hr. The experiment lasted 33 days.

We chose a 4 × 4 (four treatments, four replicates) design to study the competition between *Euplotes* and mono‐ and polyclonal *Coleps* (*Col* poly) populations, resulting in the following combinations: Treatment 1: *Euplotes* – *Col* 1, Treatment 2: *Euplotes* – *Col* 2, Treatment 3: *Euplotes* – *Col* 3, Treatment 4: *Euplotes* – *Col* poly (*Col* 1, *Col* 2, *Col* 3). In each treatment, the experimental communities were supplied with the same mixture of the microalgae *Cry* and *Nav,* added once at the beginning of the experiment. The initial total ciliate biovolume in the experimental units was 1.3 × 10^6^ µm^3^/ml, and the total microalgal biovolume was 14.4 × 10^6^ µm^3^/ml. Different ciliate and microalgal species were inoculated with equal biovolume, respectively. Culture vessels were sampled every second day using a hypodermic syringe and cannula (1.0 × 200 mm, BD Plastipak, B. Braun, Melsungen, Germany; neoLab Migge, Heidelberg, Germany). The total sample volume (60 ml) was subdivided as follows: Subsamples for microscopic analyses (30 ml) of ciliate and microalgal abundance were taken every second day. Subsamples for nutrient analyses (20 ml) were taken every fourth day. On dates without nutrient sampling, bacteria (10 ml) or subsamples for molecular biological analysis (30 ml) were taken alternating every eighth day starting with bacteria samples on day 5 and molecular samples on day 9.

### 2.3 Sample processing and analysis

Plankton samples were fixed with Lugol's solution (1% final concentration) and stored in brown glass bottles. Algal abundance was analyzed microscopically (Leica DMIL) counting at least 400 cells per sample in randomly placed squares (Lund et al., 1958) if possible. Subsample size was 0.1 ml for the highly abundant *Navicula pelliculosa* and 1–2 ml for *Cryptomonas* sp. In cases where algal abundance was too low following this method, either two 0.5 mm transects at 100× magnification in a subsample of 2 ml (equaling a sixth of the counting chamber or a subsample of 0.335 ml) or the entire subsample was counted. Ciliate abundance was counted in a subsample sized 2 ml. If no ciliates were detected, an abundance of 0.5 × the detection limit (0.25 cells/ml) was assumed (Clarke, 1998). The different cell size dimensions of 20 individuals of each ciliate and algal species were once determined using a digital image system program (Cell‐P) to calculate the average specific biovolume (Hillebrand et al., 1999). These data were used to calculate population biovolume. Initial net population growth rates were calculated according to:

$$r=\frac{\ln B_{2}‐\ln B_{1}}{t_{2}‐t_{1}}$$

where *r* denotes the net growth rate per day, *t*_1_ and *t*_2_ are two points in time, and *B*_1_ and *B*_2_ denote the population biomass at *t*_1_ and *t*_2_, respectively.

The average population filtration rate (*F*) during the initial growth phase of the experiment was estimated using:

$$F=I_{\max}*\bar{C}$$

where *I*_max_ signifies the maximum ingestion rate of the ciliates (Table 1) and $\bar{C}$ the time‐averaged ciliate density, which was calculated as follows:

$$\bar{C}=\frac{C_{2}‐C_{1}}{\ln C_{2}‐\ln C_{1}}$$

where *C*_1_ and *C*_2_ denote the population density at *t*_1_ and *t*_2_, respectively.

Bacteria samples were preserved with Glutaraldehyde (final concentration 1%). Diluted (1:15) subsamples (3 ml) were stained with DAPI (Porter & Feig, 1980), collected on black polycarbonate membrane filters (diameter: 25 mm) of 0.2 µm pore size (Whatman Cyclopore) and analyzed by epifluorescence microscopy (Axiophot, Zeiss) counting the bacteria in 10 grids at 1,000× magnification (0.1 mm^2^).

Samples for analysis of soluble reactive fractions of nitrogen, phosphorus and silicate concentrations were filtered using syringe filters (0.2 µm, cellulose acetate, Macherey‐Nagel) and stored frozen (−20°C). They were analyzed using a Scalar analytical auto‐analyzer (San^++^ System, Scalar Analytical, Breda, The Netherlands), following the methods published by Grasshoff et al. (1999).

Molecular samples were collected on glass microfiber filters (Whatman GF‐F), which were transferred to Falcon tubes and stored frozen (−80°C) until further analysis.

### 2.4 Molecular biology

#### 2.4.1 Cell disruption and DNA isolation

Samples (20 ml of cell culture) for genomic DNA analysis were collected on glass microfiber filters (Whatman GF‐F), transferred to Falcon tubes and stored frozen at −80°C. For cell disruption, 1 ml of 2× lysis buffer (40 mM EDTA; pH 8, 100 mM Tris‐HCl; pH 8, 100 mM NaCl, 1% (w/v) SDS) was added to the filtered material and swirled until the filters were completely soaked. Samples were vortexed together with 0.5 mm zirconium beads for 30s, followed by incubation at 70°C for 5 min. The procedure was repeated two times. Lysates were filtered using syringe filters and collected in fresh 15 ml centrifuge tubes. 10% CTAB solution and 2.5 M NaCl were added to the lysates and adjusted to a final working concentration of 1% and 0.7 M, respectively. Samples were incubated for 10 min at 70°C. Genomic DNA was subsequently extracted using phenol–chloroform, following Countway et al. (2007) and stored at −20°C until polymerase chain reaction (PCR) was conducted.

#### 2.4.2 Specific primer design and PCR

Earlier sequencing attempts by us, as well as studies from Pröschold et al. (2021) revealed that the 18S rRNA genes of *Coleps hirtus* clones are highly similar, and therefore not suitable to distinguish our three clones. Therefore, we used the primer pair ITS *F* (5’‐GAAACTGCGAATGGCTC‐3’) and ITS R (5’‐TTGGTCCGTGTTTCAAGACG‐3’) based on Jerome and Lynn (1997), to amplify a ~ 2.8 kb section of our clones’ genomes, consisting of the 18 S rRNA gene, ITS‐1 region, 5.8 S rRNA gene, ITS‐2 region, and partial 28 S rRNA gene. For more details on the amplification and sequencing methods of the large ~2.8 kb DNA fragment, see Supporting Information). Due to the relatively size of this PCR product, and the requirement to fulfill conditions (PCR products of ~500 bp) for the subsequent analysis with denaturing gradient gel electrophoresis (DGGE), the primers 3770F and 2104R that bind specifically to the conserved regions up‐ and downstream from the hypervariable ITS‐1 and ITS‐2 regions of the clones were designed. The sequences of 3770F and 2104R are 5’‐GAT CCG GTG AAC CTT CTG GAC‐3’ and 5’‐CGG CGC TTT ATC CTA TTT TGG C‐3’, respectively. Primer specificity and presence of potential binding targets were checked using the Primer BLAST tool from NCBI (https://www.ncbi.nlm.nih.gov/tools/primer‐blast/). All primers were synthesized by biomers.net GmbH (Ulm, Germany), and primer functionality was tested by performing a gradient PCR (Mastercycler Pro S; Eppendorf AG, Hamburg, Germany) to establish the optimum annealing temperature for the primer pair. Each reaction mix (50 µl) contained 1× PCR buffer with 2.1 mM MgCl_2_, 250 mM dNTPs, 1.5 mg/ml bovine serum albumin, 10 pmol of each primer, 1 unit of GoTaq^®^ G2 DNA polymerase (Promega Corporation, Wisconsin, USA), and 2–6 µl of template DNA. Thermocycling conditions were as follows: Initial denaturation at 95°C for 5 min, followed by 30 cycles of 1 min at 95°C, 1 min at 57°C and 3 min at 72°C, followed by a final extension for 5 min at 72°C. The resulting PCR products of the three clones (*Col* 1, *Col* 2, *Col* 3) were 510 bp, 527 bp, and 515 bp long, respectively. PCR products were purified using the peqGOLD Cycle‐Pure DNA purification kit (VWR International GmbH, Erlangen, Germany) following the manufacturer's instructions and sent to Macrogen Europe (Macrogen B.V, Amsterdam, The Netherlands) for sequencing using the primer 2104R (5 pmol/µl; 2 µl per sequencing reaction). Sequencing was performed using an automated sequencer (Applied Biosystems 3730xl DNA). Individual clones were clearly distinguishable from one another after sequence alignment of the PCR products was performed using the software “BioEdit Sequence Alignment Editor” (Hall, 1999). Intraspecific sequence variation in the ITS regions of our clones ranged between 2.7% and 4.9%. DNA sequences of our three clones have been deposited in the GenBank database under the accession numbers MW929305 (*Col* 1), MW929304 (*Col* 2), and MW929302 (*Col* 3). (Please note that the sequences deposited in GenBank for the clones are based on the ~2.8 kb ITS F/R DNA fragment mentioned above). In order for the 3770F/2104R PCR products to be analyzable with DGGE subsequently, a GC clamp adapted after Muyzer et al. (1993) was attached to the 5’end of the forward primer 3770F. The new sequence for the DGGE‐compatible primer, 3770F+GC, was as follows: 5’‐**CGC CCG CCG CGC CCC GCG CCC GTC CCG CCG CCC CCG CCCG** GAT CCG GTG AAC CTT CTG GAC‐3’ (bold nucleotides represent the attached GC clamp).

#### 2.4.3 DGGE analysis of PCR products

We used the phorU electrophoresis system (INGENY, Leiden, The Netherlands) for conducting DGGE (Fischer & Lerman, 1983), applying a specifically designed protocol for running ciliate PCR products. The PCR products were analyzed on a 15% (w/v) polyacrylamide gel, with a denaturing gradient from 5% to 60% (100% denaturant correspond to 7 mol/l urea and 40% formamide). Due to the high polyacrylamide concentration, the casted polyacrylamide gel mix was allowed to polymerize for 4 hr. Prior to loading samples onto the gel, the PCR products were mixed with loading buffer (40% [w/v] glycerol, 60% [w/v] 1× Tris‐acetate‐EDTA [TAE], bromphenol blue) at a sample/buffer ratio of 1:4. The gel was run in 1× TAE (40 mmol/l Tris, 20 mmol/l acetate, 1 mmol/l EDTA) at a constant voltage of 100 V for 24 hr and at a temperature of 60°C. After gel electrophoresis, the gel was stained in a 1× SybrGold solution (Invitrogen™ S11494, Thermo Fisher Scientific GmbH, Dreieich, Germany) for 50 min, followed by destaining in distilled water for 5 min. Finally, the gel was visualized under UV light. Differences in band migration distance allowed the identification of individual strains (see Appendix B for details).

### 2.5 Data analyses

In one of the replicates of the treatment *Euplotes*—*Col* 3, population dynamics especially of *Euplotes* differed greatly from the other three replicates (population size was up to 2.5 times higher than the average of the other replicates). Since this was due to problems concerning the flow‐through system of this particular chemostat (medium was pumped in, but culture suspension did not flow off), we removed the results for this replicate from the analysis.

We used a linear mixed model ANOVA to analyze experimental community dynamics, where the log‐ratio of *Euplotes* and *Coleps* biomass served as response variable. Treatment (combination) was a factor, and time (days) was set as trend and as factor for random fluctuations over time. The analysis was performed with R version 3.6.3 (R Development Core Team, 2020) using RStudio version 1.2.5042 (RStudio, Boston, USA).

### 2.6 Energetic and biochemical constraints

#### 2.6.1 Identifying factors limiting phytoplankton and ciliate growth

Nutrient depletion of phytoplankton was estimated from the concentrations of dissolved inorganic nitrogen in the medium. Further evidence for bottom‐up or top‐down control of the different species was obtained from the ratio between phytoplankton and zooplankton biomass, which required converting abundance measurements into carbon. We used empirically established biovolume‐to‐carbon relationships to convert from cell volume to carbon (for details, see Table 1).

#### 2.6.2 Relevance of bacteria as an additional food source for ciliates

**FIGURE 1:**
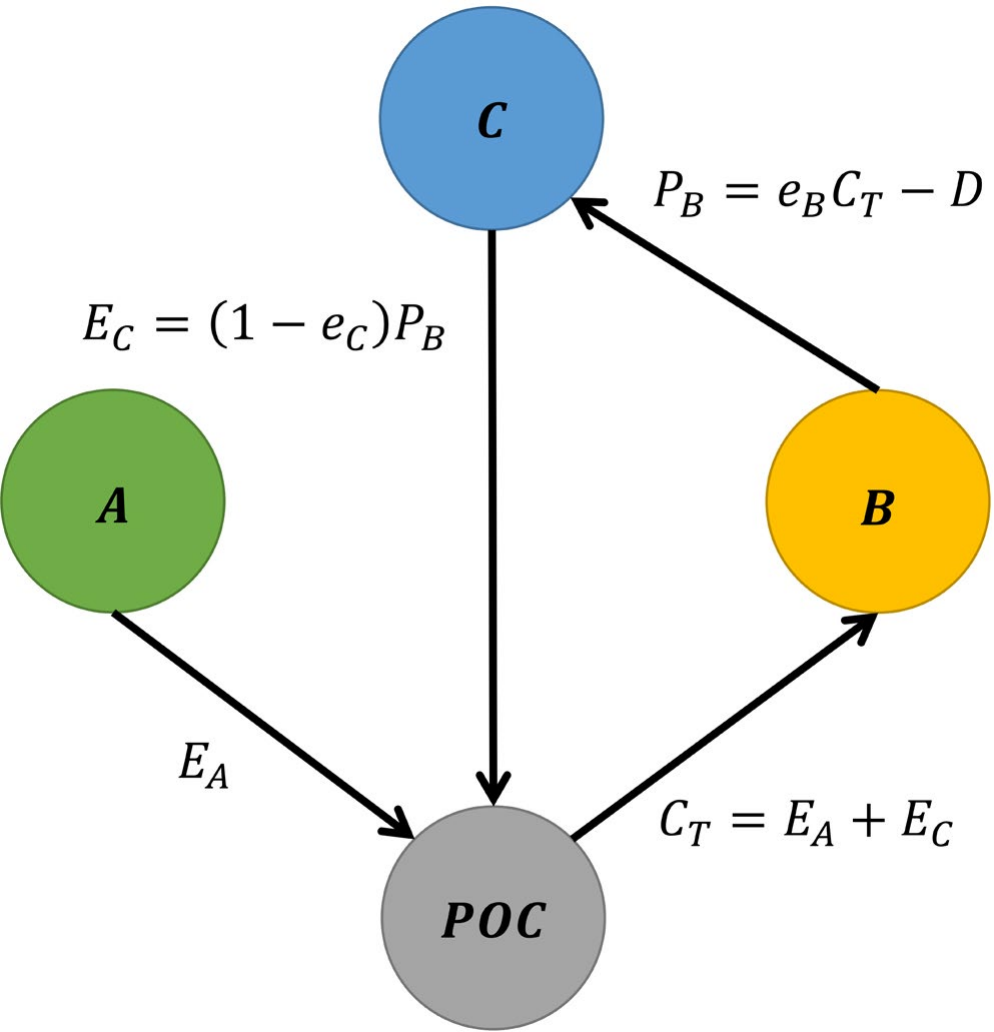
Energy flow diagram. The amount of particulate organic carbon (POC) available for the consumption (*C*_T_) by bacteria (B) depends on the exudation (*E*_A_) of the algae (A) and the excretion (*E*_C_) of the ciliates (C) (for details, see Methods section)

Since ciliates may also feed on bacteria, we estimated the potential biomass production of the bacteria, *P*_B_, in our system. *P*_B_ mainly depends on algal exudation *E*_A_ and the excretion of the ciliates *E*_C_ (Figure 1). Assuming that a fraction *f* of the gross primary production (GPP) is released through algal exudation, the net primary production (NPP) represents (1 − *f*) of the GPP. Hence, the amount of exudates is given by $E_{A}=\frac{f}{\left(1‐f\right)}\text{NPP}$. The very low temporal variability in algal densities and in the concentrations of dissolved inorganic nitrogen at the end of our experiments suggest that the phytoplankton species reached a stationary phase with approximately zero net growth. At this point, the NPP of the dominant phytoplankton species, that is, *Navicula*, has mainly to compensate for the 10% mortality through dilution because the relatively low abundance of *Coleps* suggests that additional mortality through grazing was negligible. Hence, *Nav* requires a NPP of 10% of its biomass B_N_ to compensate for its losses.

Furthermore, a part of the ingested bacteria is excreted, *E*_C_, providing an additional carbon source, resulting in a total amount of carbon available for bacterial consumption of $C_{T}=E_{A}+E_{C}$. *E*_C_ depends on the assimilation efficiency *e*_C_ of the ciliates and is, *t*s, given by $E_{C}=\left(1‐e_{C}\right)P_{B}$. Finally, *P*_B_ available for ciliates depends on the growth efficiency of the bacteria *e*_B_, and on the loss of bacterial biomass, B, through dilution *D*, that is, D=δ·B. (Figure 1). Hence, at equilibrium, *P*_B_ satisfies the following equation:

$$P_{B}=e_{B}\left(E_{A}+\left(1‐e_{C}\right)P_{B}\right)‐D.$$

Solving for *P*_B_ gives us the following expression:

(1) $$P_{B}=\frac{e_{B}E_{A}‐D}{\left(1‐e_{B}\left(1‐e_{C}\right)\right)}$$

Note, Equation (1) is equivalent with an expression derived by Raatz et al. (2018) using the geometric sum and, thus, considering the limit of a recurrent cycle of bacterial production, consumption, and subsequent partly excretion:

$$P_{B}=e_{B}E_{A}‐D+\frac{\left(e_{B}E_{A}‐D\right)e_{B}\left(1‐e_{C}\right)}{\left(1‐e_{B}\left(1‐e_{C}\right)\right)}$$

To quantify *P*_B_, we assumed *e*_B_ = 0.5 (Raatz et al., 2018) and *e*_C_ = 0.5. To compare *P*_B_ with the energetic demands of the ciliates, we estimated their maximum ingestion rate by assuming a growth efficiency of 25% and a maximum growth rate of 0.4 [1/day] based on our previous experiments.

## 3 RESULTS

### 3.1 Population dynamics of the competing ciliates and their microalgal prey

**FIGURE 2:**
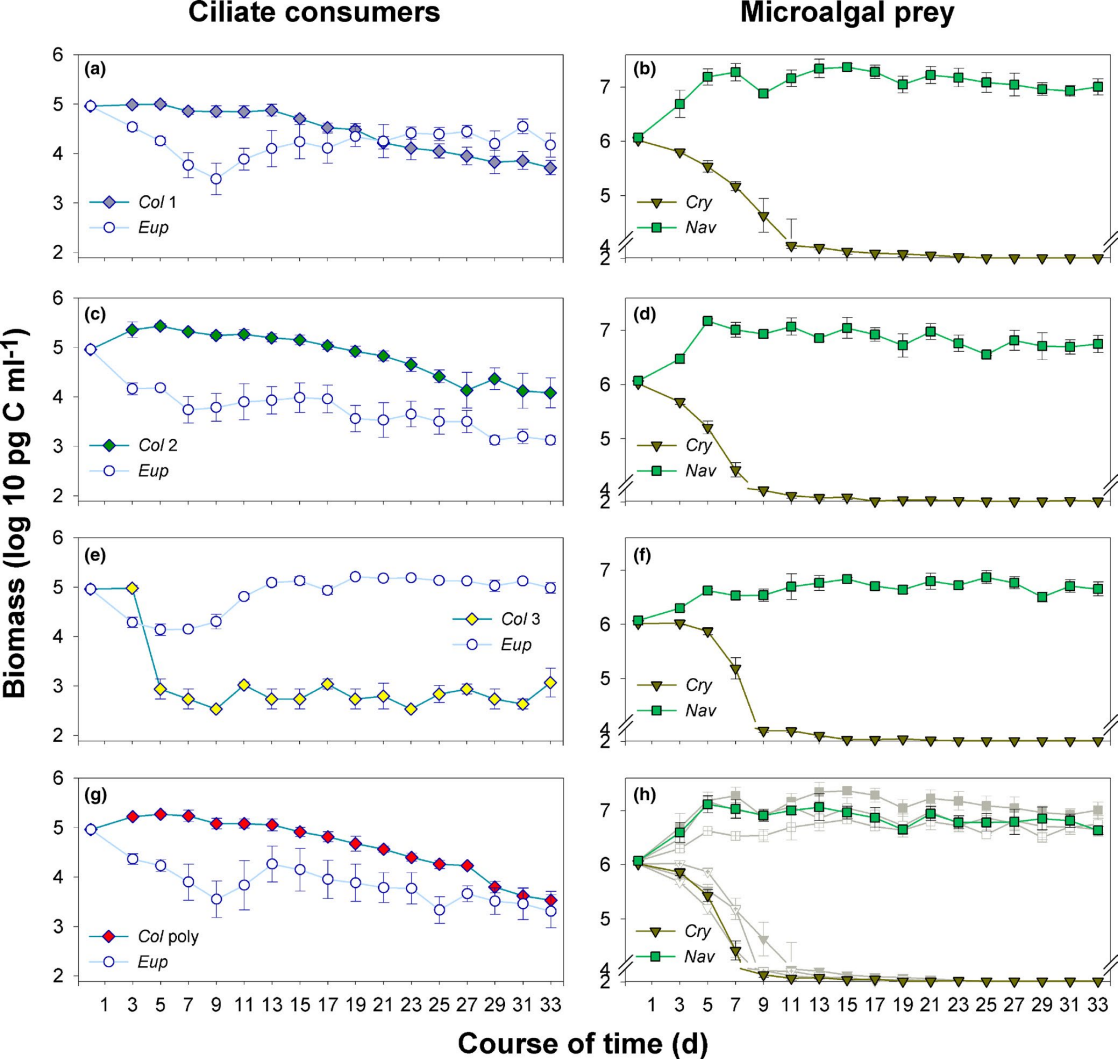
Population dynamics of ciliate predators and microalgal prey: time course of biomass, log10‐transformed. (a, b) *Euplotes*—*Col* 1, (c, d) *Euplotes*—*Col* 2, (e, f) *Euplotes*—*Col* 3, (g, h) *Euplotes*—*Col* poly. To emphasize prey trajectory differences among treatments, we added gray plots to Figure 2 h: Squares: *Navicula*, triangles: *Cryptomonas*; filled symbols: *Euplotes*—*Col* 1, open symbols: *Euplotes*—*Col* 2, crossed symbols: *Euplotes*—*Col* 3. Please note that the detection limits differ among species. They are 10^3.3^ pg C ml^−1^ for *Euplotes*, 10^2.8^ pg C ml^−1^ for all *Coleps* clones (*Col* 1, *Col* 2, *Col* 3) and 10^2.5^ pg C ml^−1^ for *Cryptomonas*. Error bars denote the standard error

Population dynamics of *Euplotes* and *Coleps* showed clear differences between the treatments (Figure 2a, c, e, and g). Either *Coleps* (*Euplotes—Col 2*) or *Euplotes* (*Euplotes*—*Col 3*) dominated at the end of the experiment, or both species still coexisted (*Euplotes—Col 1*) or equally declined (*Euplotes—Col poly*). Despite minor differences in microalgal population dynamics, fast decreasing *Cryptomonas* biomass and increasing *Navicula* biomass were a general pattern in all treatments (Figure 2b, d, f and h). Since all *Euplotes* populations went through an initial lag‐phase, this indicates that the population filtration rates of the *Coleps* clones (Table 2) could control *Cry* and but not *Nav* during this phase of the experiment.

**TABLE 2:** Net growth rate (*r*) of *Euplotes* and *Coleps*, and population filtration rate (*F*) of the *Coleps* clones (*Col* 1, *Col* 2, *Col* 3). *F _Cry_*: *F* for *Cryptomonas*, *F _Nav_*: *F* for *Navicula*. Rates are based on the initial growth phase (days 0–5), where microalgal prey density allowed for maximum ingestion rates (*I*_max_). Assuming ciliates are mostly inactive during lag‐phases, no *F* values were given for *Euplotes*. Since *Col* 3 populations initially grew, then crashed between days 4 and 5, calculating with *I*_max_ might have resulted in an overestimation of the filtration rates for this clone

Combination	*Euplotes*	*Coleps*		
	*r* d^−1^	*r* d^−1^	*F _Cry_* pg C ml^−1^ d^−1^	*F _Nav_* pg C ml^−1^ d^−1^
*Euplotes* – *Col* 1	−0.324 ± 0.035 *SE*	0.009 ± 0.014 *SE*	10^5^.^1^	10^4.5^
*Euplotes* – *Col* 2	−0.357 ± 0.022 *SE*	0.219 ± 0.021 *SE*	10^5.3^	10^4^.^3^
*Euplotes* – *Col* 3	−0.377 ± 0.062 *SE*	−0.930 ± 0.092 *SE*	10^4^.^4^	10^3.8^
*Euplotes* – *Col* poly	−0.335 ± 0.056 *SE*	0.144 ± 0.021 *SE*		

#### 3.1.1 Euplotes—Col 1

In this treatment, *Euplotes* biomass declined during the first 9 days of the experiment, then increased slightly and remained on the same level until the end of the experiment, slightly exceeding the biomass of *Col 1* in the last 10 days (Figure 2a). Following a slight upwards trend (Table 3), *Col* 1 biomass maintained a stable population density during the first 13 days, after which it started and continued declining at a rate of ca. 0.12 per day (Figure 2a), that is, similarly as the dilution rate of 0.1. *Cry* biomass in the *Euplotes*—*Col* 1 treatment declined more slowly than in other treatments, while *Nav* biomass was the highest (Figure 2b). In this treatment, both ciliate species still coexisted after competing for 33 days. If, however, the population dynamics of *Euplotes* and *Col* 1 had continued as in the final 20 days of the experiment, *Col* 1 would have eventually been excluded (Figure 2a).

**TABLE 3:** Results of linear mixed model ANOVA. Response variable: log‐ratio of *Euplotes* and *Coleps* biomass. Treatment (combination) was a factor, and time (days) was set as trend and as factor to account for random fluctuations over time

ANOVA table	*N* par	Sum Sq.	Mean Sq.	*F*‐value
Combination	3	24.7	8.22	28.7
Time	1	7.40	7.40	25.8
Combination × Time	3	13.9	4.65	16.2

Contrasts combination × time	Estimate	*SE*	*df*	*t*‐ratio	*p*‐Value
*Euplotes*—*Col* 1 < > *Euplotes*—*Col* 2	−0.88	0.31	11	−2.83	. ***0674***
*Euplotes*—*Col* 1 < > *Euplotes*—*Col* 3	2.03	0.34	11	6.06	**.0004**
*Euplotes*—*Col* 1 < > *Euplotes*—*Col* poly	−0.53	0.31	11	−1.71	.3646
*Euplotes*—*Col* 2 < > *Euplotes*—*Col* 3	2.91	0.34	11	8.68	**.0001**
*Euplotes*—*Col* 2 < > *Euplotes*—*Col* poly	0.35	0.31	11	1.12	.6853
*Euplotes*—*Col* 3 < > *Euplotes*—*Col* poly	−2.56	0.34	11	−7.64	**.0001**

#### 3.1.2 Euplotes—Col 2

In this treatment, *Euplotes* biomass declined during the first week of the experiment. After a short phase of stabilization, biomass declined further until it remained below or at the detection limit (10^3.3^ pg C ml^−1^) during the last week of the experiment. *Col* 2 biomass increased considerably during the first 5 days (Table 2, Figure 2c), then slightly decreased until day 25, and stabilized at the same level. While *Nav* biomass increased and remained on a high level, *Cry* biomass decreased fast in this treatment and reached the detection limit (10^2.5^ pg C ml^−1^) on day 13 (Figure 2d). In this treatment, *Col* 2 was able to maintain a small population, while *Euplotes* was more or less excluded (Figure 2c).

#### 3.1.3 Euplotes—Col 3

Again, *Euplotes* biomass initially decreased in this treatment until day 7, after which it increased and remained on a high level until the end of the experiment (Figure 2e). In contrast, *Col* 3 biomass increased slightly during the first three days, then the population crashed (Table 2), and was not detectable in most of the samples for the remainder of the experiment. *Cry* biomass initially declined more slowly than in the other treatments and reached the detection limit around day 15, while *Nav* biomass was lower than in the other treatments (Figure 2f). *Euplotes* was superior in this treatment (Figure 2e).

#### 3.1.4 Euplotes—Col poly

The general pattern of population dynamics in this treatment (Figure 2g) resembled the initial part of the *Euplotes*—*Col* 1 treatment and the final part of the *Euplotes*—*Col* 2 treatment (Figure 2a and c). An initial decline in *Euplotes* biomass was followed by a brief phase of net growth (Figure 2g). From day 15 to the end of the experiment, its total biomass kept declining at a rate of ca. 0.09 per day (Figure 2g). *Col* poly biomass increased during the first 5 days (Table 2), after which it declined until the end of the experiment (ca. 0.13 per day). The *Euplotes*—*Col* poly treatment displayed the fastest decline in *Cry*, which reached the detection limit on day 11 (Figure 2h). *Nav* biomass reached an intermediate level compared with the other treatments. The biomass of *Col* poly exceeded that of *Euplotes*, however, both populations kept declining and were at their detection limit by the end of the experiment, indicating that none of the species was superior to the other in this treatment (Figure 2g).

**FIGURE 3:**
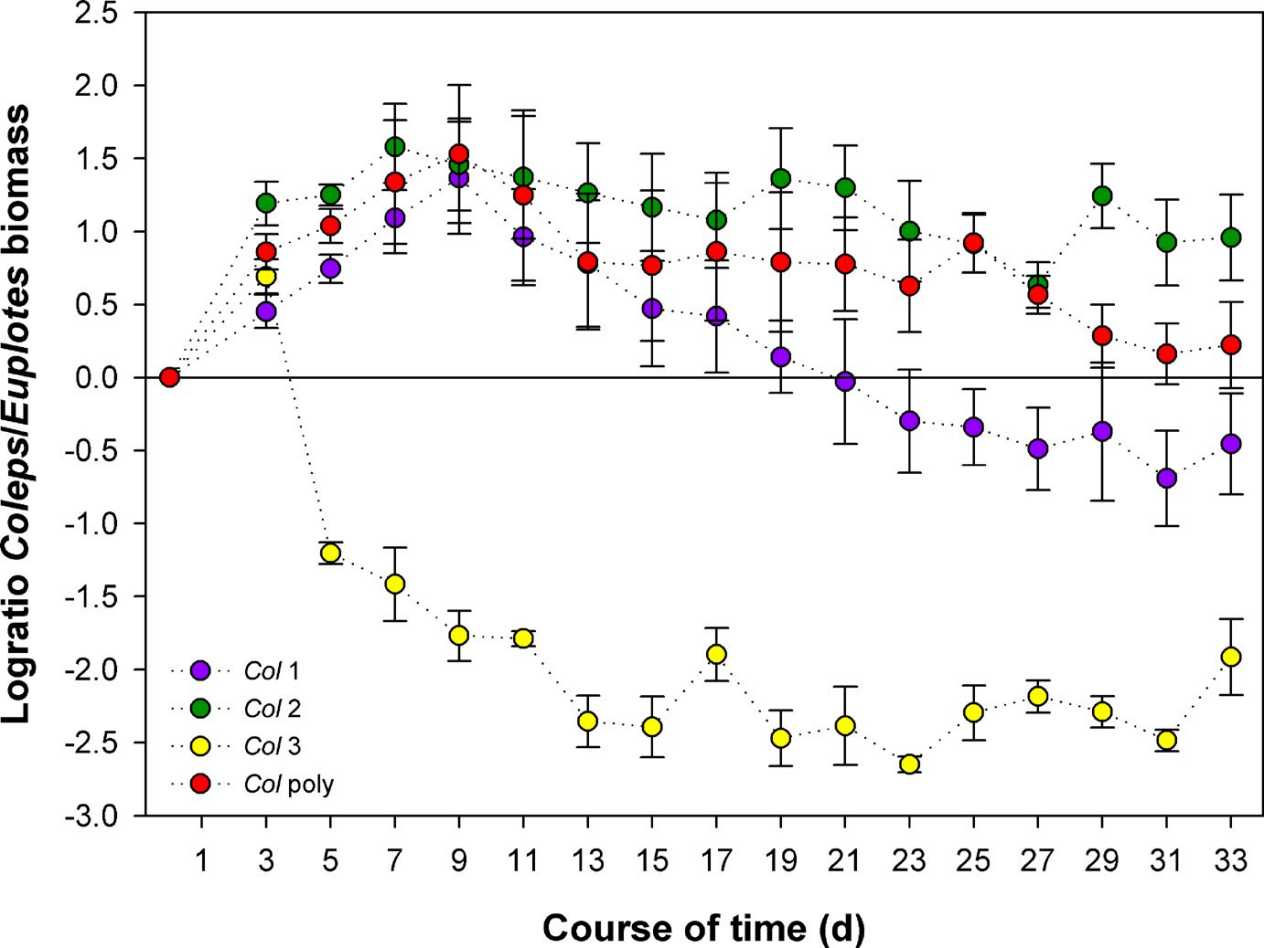
Proportion of *Coleps* to *Euplotes* biomass (BM) over the course of time, expressed as log‐ratio (log10(*Coleps* BM/*Euplotes* BM)). *Col* 1: combination *Euplotes*—*Col* 1, *Col* 2: combination *Euplotes*—*Col* 2, *Col* 3: combination *Euplotes*—*Col* 3, *Col* poly: *Euplotes*—*Col* poly. Error bars denote the standard error

The influence of the clonal composition of *Coleps* on the competition among the two ciliate species becomes even clearer when comparing their log biomass ratios (Figure 3). The log‐ratio of *Coleps* to *Euplotes* biomass was lowest in the *Euplotes*—*Col* 3 treatment, indicating a strong *Euplotes* dominance. The log‐ratio of this treatment differed significantly from those of all other treatments (linear mixed model ANOVA, highly significant contrasts; Table 3). The log‐ratio was highest in the *Euplotes*—*Col* 2 treatment, indicating *Coleps* dominance, and it was marginally significantly different from the *Euplotes*—*Col* 1 treatment (*p* < .067). The ratios of the remaining treatments (*Euplotes*—*Col 1,* *Euplotes*—*Col poly*) both declined after an initial increase and remained around 0, pointing to equal biomasses of both species. No significant difference was detected between *Euplotes*—*Col poly* and both, the treatment *Euplotes*—*Col* 1 and *Euplotes*—*Col* 2 (Figure 3, Table 3).

For the *Coleps* polyculture, DGGE revealed that only two of the three *Coleps* clones (*Col* 1 and *Col* 2) were still present on day 9 of the experiment (Appendix B). The population of *Col* 3 could not be detected anymore, indicating that it had already declined below detection limit by day 9, which corresponds to the dynamics observed in the monoclonal *Euplotes—Col* 3 treatment. On this day, the DNA band for *Col* 1 appeared to be brighter than the one for *Col* 2, indicating that this clone contributed a higher portion to the *Coleps* population than *Col* 2. On day 17, the DNA band of *Col* 2 was brighter than on day 9, while the one of *Col* 1 had faded, suggesting that *Col 2* dominated the population at the time, which is again in line with the monoclonal dynamics. On day 25, the DNA band of *Col* 1 was hardly visible anymore, while the band of *Col* 2 had also started to fade. No DNA bands were detected for day 33, which corresponds to the low *Col poly* population density at the end of the experiment (Figure 2g, Appendix B).

### 3.2 Nutrient concentrations

**FIGURE 4:**
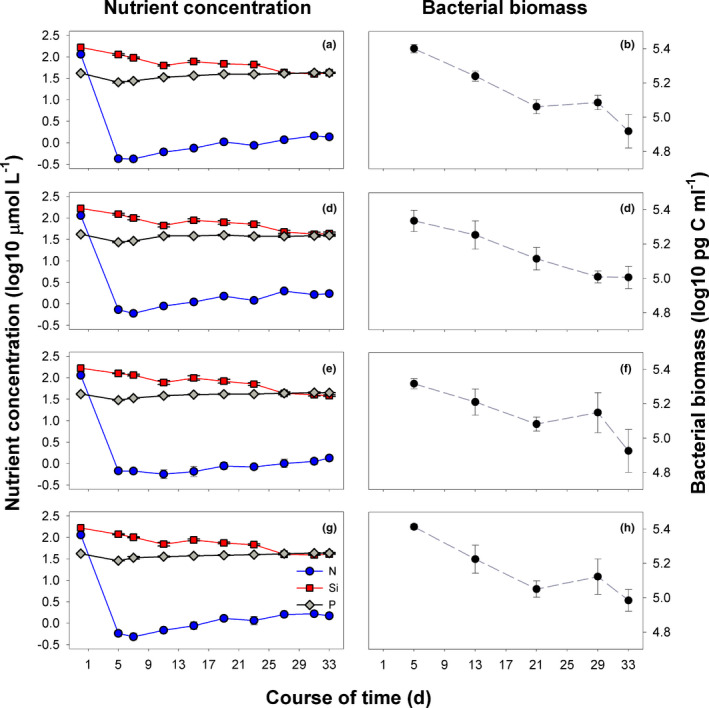
Time course of nutrient concentration (log10‐transformed) and bacterial biomass. Limiting nutrient nitrogen (N), phosphorus (P), and silicate (Si). (a, b) *Euplotes*—*Col* 1, (c, d) *Euplotes*—*Col* 2, (e, f) *Euplotes*—*Col* 3, (g, h) *Euplotes*—*Col* poly. Error bars denote the standard error

Phosphorus concentrations remained more or less constant over the course of the experiment in all treatments, while silicate concentrations slightly decreased over time, until in the last week of the experiment the decrease leveled off at concentrations around 42.3 (± 0.76 *SE*) µmol/l, suggesting that silicate never limited diatom growth (Lampert & Sommer, 2007). Concentrations of the limiting nutrient nitrogen decreased rapidly during the first 5 days of the experiment (Figure 4a, c, e, g). On day 5, N‐concentrations ranged between 0.43 µmol/l (± 0.033 *SE*) in the *Euplotes*—*Col* 1 treatment (Figure 4a) and 0.74 µmol/L (± 0.057 *SE*) in the *Euplotes*—*Col* 2 treatment (Figure 4c). In all treatments, N‐concentrations after day 5 increased slightly. However, they remained below 1.7 µmol/l, indicating severe N‐depletion. A strong bottom‐up regulation of the algae is further indicated by the very low ratio between ciliate and phytoplankton biomass, ranging only between <0.01 and 0.04 in all treatments.

### 3.3 Relevance of bacteria for food web dynamics—energetic and stoichiometric constraints

**FIGURE 5:**
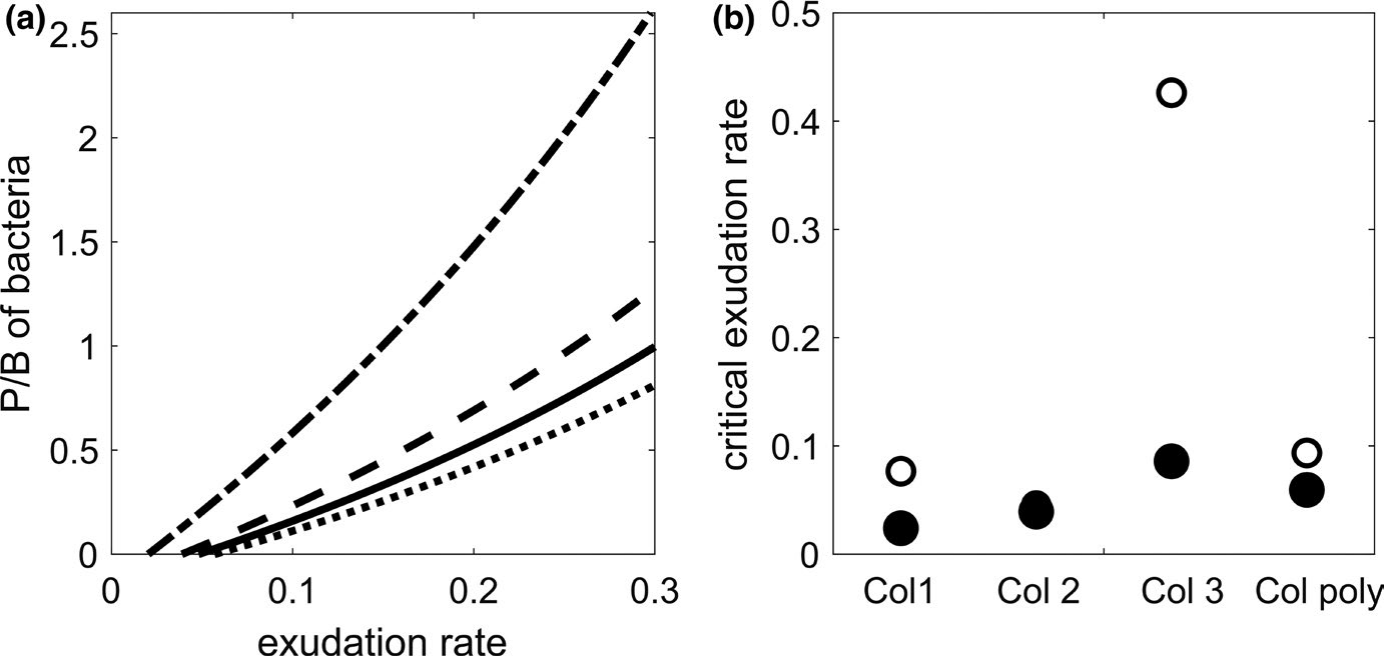
Comparison between the energy potentially supplied by bacteria to *Euplotes* and the maximum ingestion rates and losses through dilution of *Euplotes*. (a) The P/B of the bacteria in dependence of the algal exudation rate for the treatment *Col* 1 (dashed‐dotted line), *Col* 2 (dashed line), *Col* 3 (solid line), and *Col* poly (dotted line). (b) Critical exudation rate for which the bacterial production equals the maximal amount of biomass *Euplotes* can ingest (open dots) and the losses of *Euplotes* through dilution (filled dots). All calculations are based on the average cell counts of the last sampling day

Bacteria in the nonaxenic cultures developed similarly in all treatments. Highest bacterial biomass was detected on day 5 of the experiment, after which it gradually decreased until the end of the experiment (Figure 4b, d, f, and h). Assuming an upper limit for algal exudation rate of 30% of the gross primary production and that the bacteria invested 50% of the exudates into own production results in a ratio between bacterial production to biomass (P/B) below 2.6 [1/day]. This corresponds to a growth rate of up to 1.25 [1/day], which likely is below their maximum growth rate (Figure 5a). The observed bacterial biomass is thus sufficient to consume the exudates, enabling moderate P/B ratios of the bacteria (Figure 5a). However, we might have underestimated the daily supply of particulate organic carbon available for bacterial growth because dead *Navicula* could have served as an additional carbon source. Furthermore, the bacterial production could have easily sustained the observed biomass of *Euplotes* at the end of the experiment in all treatments and it was sufficient for a re‐increase in the biomass of *Euplotes* from an energetic point of view. This holds even for a moderate exudation rate between 0.05 and 0.1 which was sufficient for a bacterial production saturating the maximum ingestion rate of *Euplotes* except for Col 3 requiring a higher exudation rate (Figure 5b).

## 4 DISCUSSION

The clonal identity of the *Coleps* populations significantly affected the outcome of competition between *Coleps* and *Euplotes*. These effects, however, did not corroborate the mechanisms proposed in our hypotheses. The hypotheses were based on the assumption that the availability of additional resources is beneficial for both, *Euplotes* and the *Coleps* clones, and that the difference in trait values that characterize how much of each prey species is ingested explain differences in competitive performance, especially of the *Coleps* clones. However, *Navicula* quickly escaped top‐down control and eventually reached a state where it was of presumably low food quality for the ciliates and thus did not allow for positive ciliate net growth rates. Furthermore, the magnitude of initial ciliate grazing and growth rates determined the outcome of competition.

The low food quality of *Navicula* was indicated by the observation that populations of the clones *Col* 1, *Col* 2, and *Col* poly ceased growing after an initial phase of growth, and started decreasing when *Cry* biomass became depleted. *Nav* biomass increased within the first five days of the experiment and then remained on a high level throughout the experiment, suggesting a low grazing pressure on *Nav*. *Cry* populations did not recover. Even at low ciliate biomass, there was no sign of *Cry* re‐invading the dominating *Nav* population, which suggests that *Nav* is a better competitor for the limiting nutrient (N) than *Cry* (Tilman & Sterner, 1984). Together with high bacterial abundances, this caused all experimental communities to become severely N‐limited, indicating a strong bottom‐up control. With feeding on *Nav* being detrimental for growth, population dynamics of the *Coleps* clones was substantially dependent on *Cry*.

### 4.1 Ciliate population dynamics and the effect of intraspecific trait variation on competition

Once our experimental system had become severely nitrogen limited, the nutritional value of the microalgal prey was most likely insufficient to support population growth of the ciliates (Chen et al., 2010; Wickham & Wimmer, 2019). Cryptophytes and diatoms are rich in sterols and polyunsaturated fatty acids (Ahlgren et al., 1990; Beach et al., 1970; Dunstan et al., 1993), and therefore considered a high‐quality food source for ciliates (Skogstad et al., 1987). The severe N‐limitation that developed early in the experiment, however, most likely had a negative effect on the food quality, especially of *Nav* that kept growing under these conditions, potentially leading to high C:N ratios (e.g., Healey & Hendzel, 1980; Klausmeier et al., 2004; Lynn et al., 2000) and low contents of essential fatty acids (Ahlgren & Hyenstrand, 2003; Klein Breteler et al., 2005; Lynn et al., 2000). As ciliates have to acquire polyunsaturated fatty acids and sterols from their algal prey due to their limited synthesis ability (e.g., Boëchat & Adrian, 2005; Yang et al., 2015), low food quality likely resulted in reduced ciliate growth.

Inter‐ and intraspecific differences in the functional traits selectivity, ingestion, and growth rate determined the outcome of the competition between *Euplotes* and *Coleps*. Competitive dynamics essentially depended on the availability of *Cry* and on the amount of low‐quality *Nav* ingested by the *Coleps* clones. Despite high bacterial abundances (3.32–17.7 10^6^ cells/ml), *Euplotes* biomass initially decreased in all treatments and remained on a low level for several days before it started growing on days 9–11, when biomass of its preferred prey (*Cry*) was already low. In a short‐term experiment performed with the same organisms, *Euplotes* displayed a similar lag‐phase, while the *Coleps* clones immediately started growing (Flöder et al., 2018). This suggests that *Euplotes* and *Coleps* differ in their growth response.

*Col* 2 is a clone that feeds mainly on *Cry* rather than *Nav* (Table 1). According to our expectation (H 1), it should have been inferior, due to *Euplotes’* high grazing efficiency and its ability to use bacteria as an alternative food source. *Euplotes*, however, was the inferior species when competing with *Col* 2. Of all *Coleps* clones, *Col* 2 displayed the highest initial growth rate (*r* = 0.22 d^−1^) and gained dominance, while *Euplotes* biomass declined. The high initial growth rate resulted in a high level of biomass being produced by the *Col* 2 population. Due to the high *I*_max_ of *Col* 2 for *Cry* (Table 1), the filtration rate of the *Col* 2 population was high (*F*_cry_ = 10^5.3^ pg C ml^−1^ d^−1^) during the initial growth phase, which led to fast *Cry* depletion. By the time *Euplotes* started to grow, *Cry* biomass had already been reduced to low levels. *Euplotes* biomass subsequently remained on a low level and was nearly excluded by the end of the experiment, while *Col* 2 persisted at low density.

Our second hypothesis (H2) postulated that *Coleps* clones also feeding on the alternative prey *Navicula* will coexist with *Euplotes*. Both, *Col* 1 and *Col* 3, prey substantially and at comparable rates upon *Cry* and *Nav* (Table 1). *Euplotes* was superior when competing with *Col* 3, whereas *Euplotes* and *Col* 1 still coexisted at the end of the experiment. The initial net growth rate of *Col* 1 (*r* = 0.01 d^−1^) indicated a slight increase in population size, whereas the *Col* 3 population rapidly crashed (*r* = −0.93 d^−1^). As a consequence, *Col* 1 (*F*_cry_ = 10^5.1^ pg C ml^−1^ d^−1^) and especially *Col* 3 (*F*_cry_ = 10^4.4^ pg C ml^−1^ d^−1^) used less *Cry* than *Col* 2, which left a larger share of the *Cry* biomass for *Euplotes* to use for population growth. In combination with *Col* 3, *Euplotes* grew virtually without competition from day 5 onwards and reached a larger biomass than in combination with *Col* 1.

Ciliates are selective (Müller & Schlegel, 1999) and may pick prey items based on size, surface characteristics (Montagnes et al., 2008), and chemical cues (Roberts et al., 2011). However, there is no evidence of selectivity based solely on food quality, selecting a prey species when its nutritional value is high but rejecting the same species at low food quality (Wickham & Wimmer, 2019). It can be expected that, regardless of its nutritional value, the *Coleps* clones kept ingesting *Nav* at the rate that is specific to their genotype. As long as the abundance of both prey species allowed feeding at *I*_max_, *Nav* contributed roughly 27% of the microalgal biomass ingested by the clones *Col* 1 and *Col* 3. The proportion of *Nav* was considerably smaller (8%) in the diet of *Col* 2. These percentages likely increased following *Cry* depletion. While the high proportion of *Nav* in the diet can explain the lack of growth in *Col* 1 during the first phase of the experiment and the following steady decline of its population size, it seems unlikely that poor food quality was also responsible for the sudden crash of the *Col* 3 population. The rapid population decline that affected all replicates in a seemingly synchronized manner rather suggests that other factors might have played a role which were not assessed in this study.

Since they feed substantially on both algal species, we expected polyclonal *Coleps* populations to coexist with *Euplotes* (H3). Superior to monoclonal populations due to higher trait variation, we expected polyclonal *Coleps* populations to exploit available microalgal prey more effectively and thus to produce high biomass levels. The initial growth rate of *Col* poly (*r* = 0.14 d^−1^) was lower than the one of *Col* 2 and much higher than the growth rates of *Col* 1 and *Col* 3, and biomass of *Col poly* remained below the one of the best performing monoculture (*Col* 2). According to the newly developed DGGE assay to distinguish the different *Coleps* clones, *Col* poly consisted of only *Col* 1 and *Col* 2 early on in the experiment. Initially, *Col* 1 contributed a large proportion to the *Col* poly population; however, *Col* 2 gained dominance halfway through the experiment. Remarkably, the *Cry* trajectory initially resembled the one observed the *Euplotes*—*Col* 1 treatment and later one of the *Euplotes*—*Col* 2 treatment. By the time *Euplotes* started to grow in this treatment, *Cry* biomass had been reduced to a much lower level than in the treatment where *Euplotes* successfully competed with *Col* 1. Accordingly, *Euplotes* biomass remained on a low level. When N became severely limiting causing the algal food quality to deteriorate and *Cry* biomass reached a low level, both, *Euplotes* and *Col* poly biomass, started and continued decreasing. Flöder et al. (2018) demonstrated transgressive overyielding based on clone‐specific differences in feeding niches (see Fridley, 2001; Tilman et al., 1997) for the same clones used in the present study. In this experiment, however, low food quality likely reduced the potential for complementarity and thus overyielding.

In contrast to Flöder et al. (2018), we were able to distinguish the different *Coleps* clones using the ITS region on their ribosomal DNA. While the 18S rRNA gene is a frequently used marker for ciliate phylogeny, these sequences are highly conserved caused by a strong selection against any loss‐of‐function mutation in the ribosome subunit gene (Barth et al., 2008; Poczai & Hyvönen, 2010). It did not prove to be suitable for assessing intraspecific sequence variation in the *Coleps* clones used in our study. Several other nuclear and mitochondrial genomic markers have been used with varying success to assess inter‐ and intraspecific sequence variations between ciliates (Barth et al., 2008; Zhao et al., 2013), including ITS regions (Diggles & Adlard, 1997; Li et al., 2017). Our ITS‐based assay was crucial for our understanding of the population dynamics in the *Coleps* polyculture, which could be nicely matched with the clonal behavior in monoculture.

Overall, this study revealed that variation in selectivity, ingestion, and growth rates, and the availability and nutritional value of the microalgal prey may explain ciliate dynamics. Phases of population growth in *Euplotes* coincided with *Cry* rather than the availability of bacteria, as the latter were highly abundant in all treatments. In the *Euplotes*—*Col* 1 and *Euplotes*—*Col* 3 treatments, however, *Euplotes* was able to maintain its population size after its preferred food source *Cry* was depleted. This suggests that the bacterial production was sufficient to maintain the population of *Euplotes* toward the end of our experiment. Bacterial production may have even allowed a further increase in the biomass of *Euplotes* but its biomass remained rather low. This may be explained by the generally rather low food quality of bacteria. Given the very low abundance of the preferred high‐quality algal prey *Cry* during most of the time in our experiment, the ciliates may have lacked essential components in their diet such as sterols, which negatively affected their population growth (cf. Raatz et al., 2017, 2018). This is in line with the observation that cultures of *Euplotes* can be sustained with a wheat grain and bacteria but generally grow better with algae.

## 5 CONCLUSION

Our study demonstrates that trait variation at both trophic levels codetermined the outcome of consumer competition in our experiment. The strength of interspecific competition strongly depended on clone‐specific differences in growth and grazing rates among the different consumers. We observed strong selection on the consumer traits in the polyclonal culture of Coleps. A novel PCR‐DGGE approach developed for the distinction of different Coleps clones enabled us to follow clonal sorting in the polyculture, an important process determining the extent of the potential trait variation in a system in addition to species sorting and phenotypic plasticity. Hence, this DGGE assay can be applied in similar future studies investigating the ecology and dynamics of clonal populations.

Unexpectedly, additional changes in the prey's food quality turned a previously advantageous consumer trait into a disadvantageous one, showing that trait values may be beneficial in one setting and disadvantageous in another, which suggests that the resulting effects are context dependent. Via context dependency, intraspecific variation might ensure the overall fitness of a species in variable and changing environments, thus contributing to community stability.

## CONFLICT OF INTEREST

None declared.

## AUTHOR CONTRIBUTIONS

**Sabine Flöder:** Conceptualization (equal); Formal analysis (lead); Investigation (lead); Methodology (equal); Writing‐original draft (lead). **Joanne Yong:** Investigation (equal); Methodology (equal); Writing‐original draft (supporting). **Toni Klauschies:** Conceptualization (equal); Formal analysis (equal); Funding acquisition (equal); Writing‐review & editing (equal). **Ursula Gaedke:** Conceptualization (equal); Methodology (equal); Writing‐review & editing (equal). **Tobias Poprick:** Investigation (equal); Methodology (supporting). **Thorsten Brinkhoff:** Methodology (equal); Resources (supporting). **Stefanie Moorthi:** Conceptualization (equal); Funding acquisition (equal); Writing‐review & editing (equal).

## Figures and Tables

**FIGURE A1:**
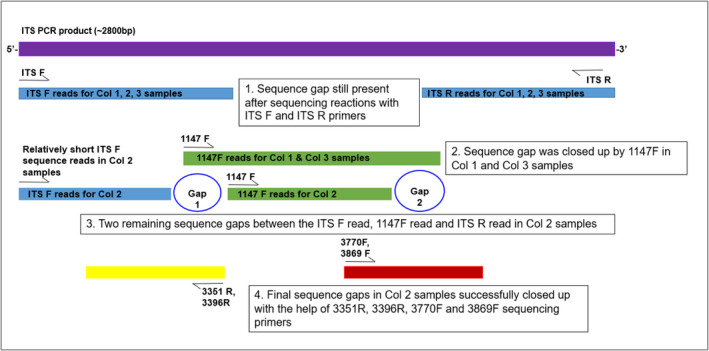
Schematic diagram of the respective sequencing reactions using various primers (1147F, 3351R, 3396R, 3370F and 3869F) for closing the two sequence gaps of the sequence of the ITS F/R PCR fragment of the *Coleps hirtus* clone *Col* 2

**FIGURE A2:**
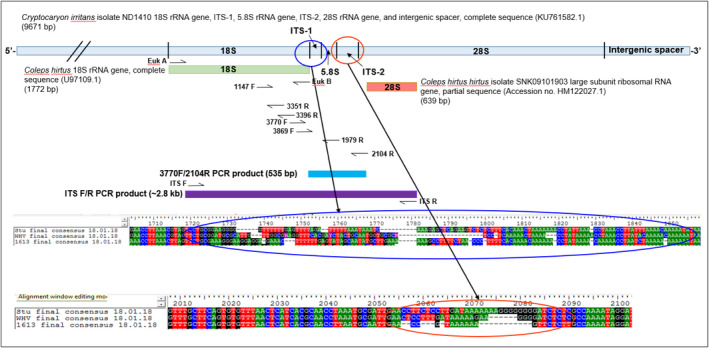
Schematic diagram of the almost completely sequenced rRNA operon of the three *C*. *hirtus* clones. All PCR and sequencing primers, PCR product sizes, binding regions as well as reference sequences for sequence alignments are illustrated. Most of the sequencing reactions were performed on the ~2.8 kb SS‐LS PCR fragment. In addition, the diagram includes zoomed up sections of the ITS‐1 (blue circle) and ITS‐2 (red) regions showing significant differences in sequence between the seven *C*. *hirtus* clones, including insertions and deletions. Legend: Blue bar represents the reference sequence from *C*. *irritans*, green bar the 18S rRNA gene sequence of *C*. *hirtus* and orange bar the 28S rRNA gene sequence of *C*. *hirtus hirtus*

**FIGURE A3:**
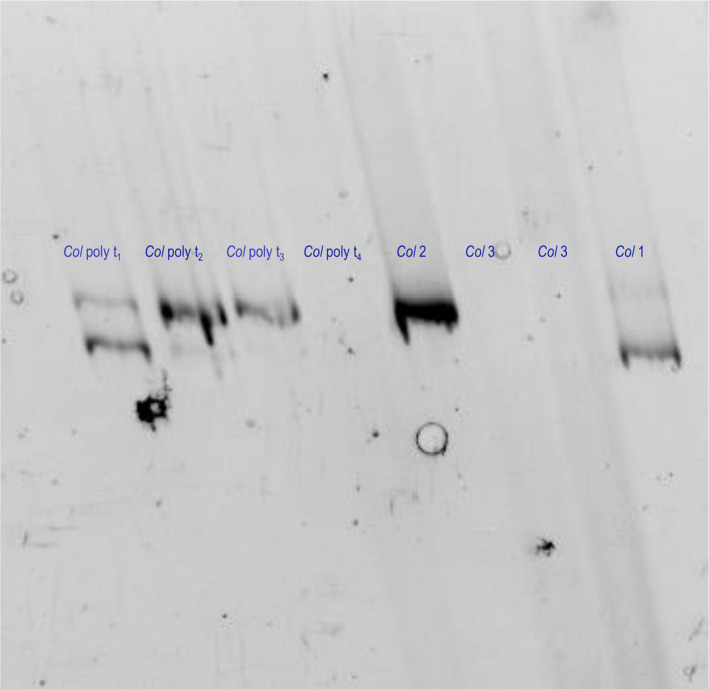
DGGE result. *Coleps* (*Col*) polyculture results are from day 9 (t1), day 17 (t2), day 25 (t3) and day 33 (t4). As references we used monoculture DNA extracts from day 9 (t1), when population density was still high in all treatments except the treatment *Euplotes* – *Coleps* 3. Since the population of *Col* 3 crashed early in the treatment DNA we used extracts for the DGGE

## Data Availability

Experimental data and R‐script are available on Dryad: Floeder, Sabine et al. (2021), Intraspecific Trait Variation Alters the Outcome of Competition in Freshwater Ciliates, Dryad, Dataset, https://doi.org/10.5061/dryad.hdr7sqvj8. DNA sequences of the *C*. *hirtus* clones are available at GenBank database under the accession numbers MW929305 (*Col* 1), MW929304 (*Col* 2), and MW929302 (*Col* 3).

## APPENDIX A

### Determination of maximum ingestion rates for *Coleps hirtus* clones and *Euplotes octocarinatus*

The determination of the trait value maximum ingestion rate (*I*_max_) for *Euplotes octocarinatus* and the *Coleps hirtus* clones are based on the data set published in Flöder et al. (2018) and represent monospecific cultures. Following Frost (1972) and Heinbokel (1978) growth rates (µ), grazing rates (*G*) and ingestion rates (*I*) were calculated for *Cryptomonas* sp. (*Cry*) and *Navicula pelliculosa* (*Nav*).

Growth rates (*µ*) for the microalgal prey *Cry* and *Nav* were calculated from grazer‐free control treatments:

$$\mu =\frac{\ln P_{2}‐\ln P_{1}}{t_{2}‐t_{1}}$$

Ciliate grazing rate (*G*) was calculated as:

$$G=\mu ‐\frac{\frac{\ln P_{2}}{\ln P_{1}}}{t_{2}‐t_{1}}$$

where *t*_1_ and *t*_2_ are two points in time, and *P*_1_ and *P*_2_ the population density at *t*_1_ and *t*_2_, respectively.

The time‐averaged concentration of the microalgal prey $\left(\bar{P}\right)$ in the presence of ciliates was calculated following:

$$\bar{P}=P_{1}\left[\frac{e^{\left(\mu ‐G\right)\left(t_{2}‐t_{1}\right)}‐1}{\left(t_{2}‐t_{1}\right)\left(\mu ‐G\right)}\right]$$

The time‐averaged ciliate concentration $\left(\bar{C}\right)$ was calculated as follows:

$$\bar{C}=\frac{C_{2}‐C_{1}}{\ln C_{2}‐\ln C_{1}}$$

where *C*_1_ and *C*_2_ denote the population density at *t*_1_ and *t*_2_, respectively.

The ciliate filtration rate (*F*) per individual and unit time is then calculated as:

$$F=\frac{G}{\bar{C}}$$

Ciliate ingestion rates (*I*) per individual and unit time are calculated:

$$I=\bar{P}*F$$

*I*_max_ was then estimated based on Michaelis‐Menten, assuming a half saturation constant (*k*) of 2000 *Cry* cells/m, and correspondingly 13,280 *Nav* cells/m for all *Coleps* clones. *P* signifies the prey concentration.

$$I=\frac{I_{\max}P}{k+P}$$

We chose the trait value *I*_max_ to express clonal differences in the feeding capacity of *Coleps*. Due to the interdependence of *k* and *I*_max_, however, it is not possible to determine based on our data set, if these differences are due to *I*_max_, *k* or if they are a function of both. Our *I*_max_ values are in line with Massana et al., (1996), who determined an *I*_max_ of 495 µm^3^ ind^−1^ hr^−1^ for *Coleps hirtus* feeding on a *Cryptomonas* species. This corresponds to 18 *Cry* cells (sized 664 µm^3^) per ciliate and day. For *E*. *octocarinatus*, we assumed that *I* determined for *Cry* equalled *I*_max_. This assumption is based on uptake experiments with the slightly larger *Euplotes mutabilis* (Wilks & Sleigh, 1998), where *I*_max_ (3,590 µm^3^ ciliate^−1^ hr^−1^) for large (10 µm^3^) microspheres was reached at a particle concentration of 10^4^ ml^−1^. The time‐averaged *Cry* concentration of *E*. *octocarinatus* treatments in Flöder et al. (2018) exceeded this threshold (2.4 10^4^ cells/ml).

**TABLE A1:** Grazing rates (*G*), ingestion rates (*I*) and maximum ingestion rates (*I*_max_) of *Euplotes octocarinatus* and the *Coleps hirtus* clones used in this experiment. *G*, *I* and *I*_max_ for *Cryptomonas* sp. (*Cry*) and *Navicula pelliculosa* (*Nav*) are based on the data set published in Flöder et al. (2018) and represent monospecific cultures. Note that when adjusted for the difference in ciliate biovolume, *I*_max_ of *E*. *octocarinatus* is 1.72 fold the average *I*_max_ of the *Coleps* clones

*Species*/Clone	*Cryptomonas*			*Navicula*		
	*G* d^−1^	*I* cells ciliate^−1^ d^−1^	*I*_max_ cells ciliate^−1^ d^−1^	*G* d^−1^	*I* cells ciliate^−1^ d^−1^	*I*_max_ cells ciliate^−1^ d^−1^
*Col* 1	0.179 ± 0.013	16.2 ± 0.9	17.6 ± 0.44	0.167 ± 0.015	17.3 ± 1.2	38.8 ± 0.51
*Col* 2	1.162 ± 0.011	3.0 ± 0.07	18.6 ± 0.31	0.037 ± 0.007	8.8 ± 1.3	12.1 ± 1.89
*Col* 3	0.111 ± 0.009	15.3 ± 0.5	17.0 ± 0.64	0.182 ± 0.055	16.8 ± 1.0	35.1 ± 4.98
*Euplotes*	0.080 ± 0.017	116 ± 5.4	116 ± 5.4	0.000 ± 0.015	—	—

## APPENDIX B

### Amplification and sequencing of the partial 18 S rRNA gene, ITS‐1, 5.8 S rRNA gene, ITS‐2 and partial 28 S rRNA gene, based on Jerome and Lynn (1997)

To amplify the ~2.8 kb DNA fragment comprising of the almost entire 18 S rRNA gene, ITS‐1 region, 5.8 S rRNA gene, ITS‐2 region and the partial 28 S rRNA gene, the primer pair ITS *F* (5’‐GAAACTGCGAATGGCTC‐3’) and ITS R (5’‐TTGGTCCGTGTTTCAAGACG‐3’) were used. Each PCR reaction mix contained 2–6 µl of template DNA (depending on the clone), 1 × PCR buffer with 2 mM MgCl_2_, 200 µM dNTPs, 0.75 mg/ml bovine serum albumin, 10 pmol of each primer, 5 units of GoTaq^®^ G2 DNA polymerase (Promega Corporation, Wisconsin, USA) in a total volume of 50 µl. Thermocycling conditions were as follows: Initial denaturation at 95°C for 5 min, followed by 35 cycles of 1 min at 95°C, 1 min at 55°C and 90s at 72°C, followed by a final extension for 5 min at 72°C. DNA extract from the ciliate *Stylonychia* sp. was used as a positive control. PCR products were purified and prepared for sequencing as described in the methods section, with the exception that the PCR products were sequenced with the ITS F and ITS R primers.

### Sequence alignment of ITS F and R sequenced PCR products

The 18S rRNA gene sequence of *Coleps hirtus* (NCBI Accession no. U97109.1), and the 28S rRNA gene sequence of *Coleps hirtus hirtus* isolate SNK09101903 (NCBI Accession no. HM122027.1) were used as reference sequences for the alignment of our sequenced PCR products. Due to the absence of long *Coleps hirtus* sequences in the NCBI Nucleotide database that span the 18S rRNA gene up to the 28S rRNA gene, a ~ 9.6 kb complete sequence of *Cryptocaryon irritans* isolate ND1410 (Accession no. KU761582.1) that possessed sufficiently high sequence similarity in the 18S and 28S regions to *Coleps hirtus* was used instead. This sequence comprises of a segment of genomic DNA, the 18S rRNA gene, ITS‐1, 5.8S rRNA gene, ITS‐2, 28S rRNA gene, and the intergenic spacer, and served as a general scaffold for the alignment of both 18S and 28S rRNA segments and to assist in mapping the almost entire rRNA operon of *Coleps hirtus*.

### Supplementary sequencing reactions with additional primers

The alignment of the ITS F and ITS R sequenced reads showed that the sequencing reactions were unable to completely sequence the entire length of the ~2.8 kb PCR fragment. All three clones still have a considerably large gap (855–1216 bp) in their sequences. Thus, the ciliophora‐specific primer 1147F (5’‐GAACGAAAGWTARGGGATCA‐3’) developed by Dopheide et al. (2008), was used as an additional sequencing primer to attempt to close up the sequence gap.

Sequence gaps in the PCR products from *Col* 1 and *Col* 3 clones were successfully closed up after sequencing using the 1147F primer. However, there were still two sequence gaps in the sequenced PCR product of *Col* 2 clones. Hence, 4 additional primers (3770F, 3869F, 3351R and 3396R) were designed to close up these two gaps within *Col* 2’s sequence. The sequences of the primers 3770F, 3869F, 3351R and 3396R are 5’‐ GATCCGGTGAACCTTCTGGAC‐3’, 5’‐CGTAGGTGAACCTGCGGAAG‐3’, 5’‐CTCAATCTGTCAATCCCACCCATG‐3’, and 5’‐CAACTAAGAACGGCCATGCAC‐3’, respectively. For an overview of the sequencing/sequence assembly process, all sequencing primers used, their binding positions, and the areas where sequence gaps were initially present but subsequently closed up, see Figure A1. During the entire sequencing and sequence assembly process, it was ensured that a particular section of the *Coleps hirtus* rRNA operon of each clone was sequenced by at least 2 different primers for 2–3 times to ensure repeated coverage and sequence accuracy. The result of our sequence assembly with multiple reads with overlapping regions, resulted in consensus sequences of the *Col* 1, *Col* 2 and *Col* 3 clones, that were 2,809 bp, 2,830 bps and 2,823 bp in length, respectively. In addition to the acquired consensus sequences of the three clones, unique and previously unknown sequences of the ITS‐1 and ITS‐2 regions of our three *C*. *hirtus* clones, were also obtained for the first time (Figure A2).
